# A hybrid analytical and data driven framework for optimizing radially grooved wet clutch geometry

**DOI:** 10.1038/s41598-025-26630-9

**Published:** 2025-11-27

**Authors:** Mohammad Sadafi, Amir F. Najafi, Alireza Jalali

**Affiliations:** School of Mechanical Engineering, College of Engineering, University of Tehran, P.O. Box 11365/4563, Tehran, Iran

**Keywords:** Wet clutch, CFD, Drag torque, Aeration, Artificial neural network, Genetic algorithm, Engineering, Mathematics and computing

## Abstract

In wet clutch systems, the drag torque is generated by the relative motion between rotating disks in the presence of the oil film. This study aims to optimize the geometry of radially grooved wet clutches to minimize drag torque. A recently developed analytical model was used to efficiently generate the required dataset in both the single- and multiphase flow conditions, as numerical simulations are computationally expensive. The accuracy of the model was first validated with CFD, showing strong agreement with a maximum deviation of 8%. Two artificial neural networks were then trained on the generated dataset and exhibited reliable performance. In the following, these data-driven models were used in an optimization study across four distinct design cases using the genetic algorithm. Numerical simulation of the fluid flow in optimized geometries confirmed significant drag torque reductions of at least 70% across all cases, with the most substantial improvement observed in Case 3 where peak drag torque decreased from 0.92 to 0.022, representing a 97% reduction. Ultimately, a parametric study was conducted to interpret the optimization results. The results showed that changing the distance between the two disks had the most significant impact on drag torque, reducing the peak value by 86%, while the groove angle had the smallest effect, with only a 2% reduction.

## Introduction

Wet clutches are important components in transmission systems, including automatic and manual types, as well as in industrial gearboxes and limited-slip differentials. When two clutch disks disengage, power transmission from the engine to the gearbox ceases, resulting in relative motion between the disks. As rotational speed increases, drag loss occurs due to the high viscosity of the oil between the disks. This drag loss accounts for a significant proportion of total transmission system losses, reaching up to 30% in some cases. This emphasizes the importance of investigating methods to reduce such drag losses, as doing so could substantially improve overall transmission efficiency.

The characteristic parameter of energy loss in wet clutch systems is the drag torque. Previous studies^1–3^ have demonstrated that the drag torque increases with rotational speed until reaching a critical threshold. Beyond this threshold, the drag torque decreases significantly due to air entry into the clutch from the outer radius. This air penetration—termed aeration—makes a two-phase flow in the clutch. Regarding the low viscosity of the air, the drag torque decreases. A detailed physical description of aeration is provided in Ref.^4^. The rotational speed at which aeration initiates is defined as the *critical speed*. Consequently, aeration represents a useful phenomenon in wet clutch systems, and lowering the critical velocity accelerates the aeration.

It has been well established that radially grooved wet clutches exhibit improved performance in reducing the drag torque, primarily by facilitating oil discharge and promoting earlier aeration^5^. As a result, grooved designs generally lead to lower overall drag losses compared to non-grooved clutches. To further enhance clutch performance, it is essential to optimize the groove geometry, as the design of the grooves significantly influences the flow behavior and resulting drag torque.

Aphale et al.^6^ performed an experimental and numerical study on the grooved wet clutch. They considered the simple radial, inclined, and hourglass groove patterns. Their numerical results were in accordance with experiments; the inclined groove pattern caused a more reduction in the drag loss than the other pattern. Cui et al.^7^ investigated the governing equations of the fluid in wet clutches and validated their findings through experimental results. Additionally, they proposed an analytical model to predict the drag torque. Wu et al.^8^ have built up an experimental system for flow pattern identification inside the radially grooved wet clutches and studied the flow transition from one-phase to two-phase condition. Wu et al.^9^ studied the fluid flow inside the wet clutch numerically and used the experimental data from their previous work to validate the numerical results. They focused on the groove number effect on the drag torque and proved that the drag torque decreases as the groove number increases. Pahlovy et al.^10^ represented a set of analytic equations to predict the drag torque and critical speed for a radially grooved wet clutch. While the model indicated satisfactory results in the single-phase condition, it had considerable deviations from experiment in the two-phase condition. Neupert et al.^11^ carried out several experiments to study how different parameters affect the drag torque. Later, they combined experiments and simulations to examine pressure distribution across the clutch and the resulting axial forces on the disks^12^. Goszczak et al.^13^ studied on a clutch used in an automatic transmission and focused on the effect of oil flow rate, gap between two disks and oil temperature on the drag torque value. Leister et al.^14^ simplified the flow equation for the fluid flow inside a wet clutch using an order of magnitude analysis and presented a new dimensionless number that can estimate the critical speed. Additionally, they developed a new analytical model to estimate drag torque in radially grooved wet clutches, utilizing the concept of hydraulic diameter. In a more recent work^15^, they used the defocusing particle tracking velocimetry (DPTV) method to observe the fluid flow behavior in the grooved wet clutch. They could provide a comprehensive insight into the flow patterns in the groove region when the flow is single-phase. Wang et al.^16^ introduced two new groove patterns to optimize the simple radial groove pattern performance on the drag torque reduction, namely single-throat and double-throat groove patterns. Numerical results showed that the new patterns reduced the drag torque by 25% and 48% compared to the simple radial groove pattern. Sax et al.^17^ used the DPTV method to measure the velocity field in the different sections of the wet clutch for six groove patterns and validate the computational fluid dynamics (CFD) results accordingly. They discussed some characteristics of the flow topology such as vortex forming in the groove. Sadafi et al.^18^ extended the analytical model proposed by Leister et al.^14^, modifying it to predict the drag torque under two-phase flow conditions. The modified model’s accuracy was validated through comparisons with both the laminar and the direct numerical simulation (DNS) results. Pointner-Gabriel et al.^19^ conducted experimental studies on oil flow and drag torque in deep-lubricated clutches, providing valuable test data for cases with this lubrication type.

Preceding studies have examined different methods like analytic models, CFD, and experiments to achieve a thorough insight into the drag torque behavior in the wet clutch fluid flow. Experiments indicate the most accurate results, but it takes a lot of time and cost to build up a test rig and perform several tests to gather the required data. This challenge stands out more when it is required to study the effect of different geometrical parameters which needs a lot of prototypes and increases the number and costs of the test. CFD can provide detailed information on each parameter at the desired time and location. However, CFD simulations entail substantial computational and time-related expenses.

A large number of preceding studies have investigated the drag torque behavior in clutches with different groove patterns, but limited attention has been paid to the influence of groove geometry (e.g., depth, width, or curvature) on drag torque and aeration. These works predominantly emphasize the presence of grooves rather than their specific geometric characteristics. However, systematic analysis of geometric parameters and optimizing them can yield substantial improvements in the drag torque value. Nevertheless, research focusing on optimization strategies for wet clutches remains limited.

Machine learning techniques have emerged as powerful tools for estimating complex physical parameters that are difficult to measure experimentally or model analytically. These data-driven models offer fast and accurate predictions, even in scenarios where traditional methods are inadequate. Approaches such as regression, artificial neural networks (ANNs), and ensemble algorithms can efficiently handle multi-parameter datasets, capturing non-linear relationships with low computational cost. These models are especially valuable in optimization studies, where they serve as objective functions that represent the relationships between multiple design parameters.

Limited studies in the literature have applied data-driven models to analyze fluid flow in wet clutches. Zhang et al.^20^ developed a hybrid model mixing an existing analytical model^21^ and a particle swarm optimization-back propagation (PSO-BP) neural network to predict the drag torque in a radially grooved wet clutch. Their results were in good accordance with experimental data. However, the study focused exclusively on well-established parameters—such as oil flow rate and temperature—which have been extensively researched, thereby limiting the novelty of their investigation. Pointner-Gabriel et al.^22^ developed a methodology to create a data-driven model to predict the drag loss in the wet clutch and provided a more detailed description of the model and results in their subsequent work^23^. Using measurements obtained from a test rig^24^, they built a comprehensive dataset and employed a machine learning algorithm to uncover the underlying relationships between the parameters influencing the drag loss. The model ultimately predicts shear stress as a function of rotational speed. The results of this study are a step forward in the estimation of the drag loss. However, the study focused on grooved wet clutches without analyzing the impact of different groove patterns or geometric parameters. Additionally, the machine learning methodology lacked detailed explanation.

This study aims to optimize the geometry of a radially grooved wet clutch to minimize the drag torque and critical speed. Unlike earlier approaches—such as those by Zhang et al.^20^ and Pointner-Gabriel et al.^22^—which were mainly limited to predicting performance outcomes, the present work advances the field by explicitly incorporating multiple groove geometry parameters (depth, width, angle, spacing, and radial dimensions) into the optimization framework. The key contribution of this study is its step forward from prior works by integrating data-driven predictive models with optimization techniques to construct actual optimized geometries rather than providing predictions alone. To achieve this, a suitable database is first required to construct a data-driven model that serves as the objective function for optimization. Due to the high computational cost and time requirements of numerical simulations, the analytical model proposed by Sadafi et al.^18^ was employed to efficiently generate a comprehensive database. Two artificial neural networks were developed based on the generated dataset to predict the drag torque. The models demonstrated strong accuracy, supported by favorable regression metrics. The first ANN takes six geometric parameters as input, while the second uses seven; both output the corresponding drag torque. These trained models were then integrated into an optimization framework based on the genetic algorithm to identify the optimal clutch geometry. The performance of the optimized design was evaluated through well-validated CFD simulations, exhibiting significant reductions in both the drag torque and critical speed. Finally, to further interpret the optimization results, the influence of geometric variations on the drag torque was analyzed using numerical simulation data.

## Geometrical description

**Fig. 1:**
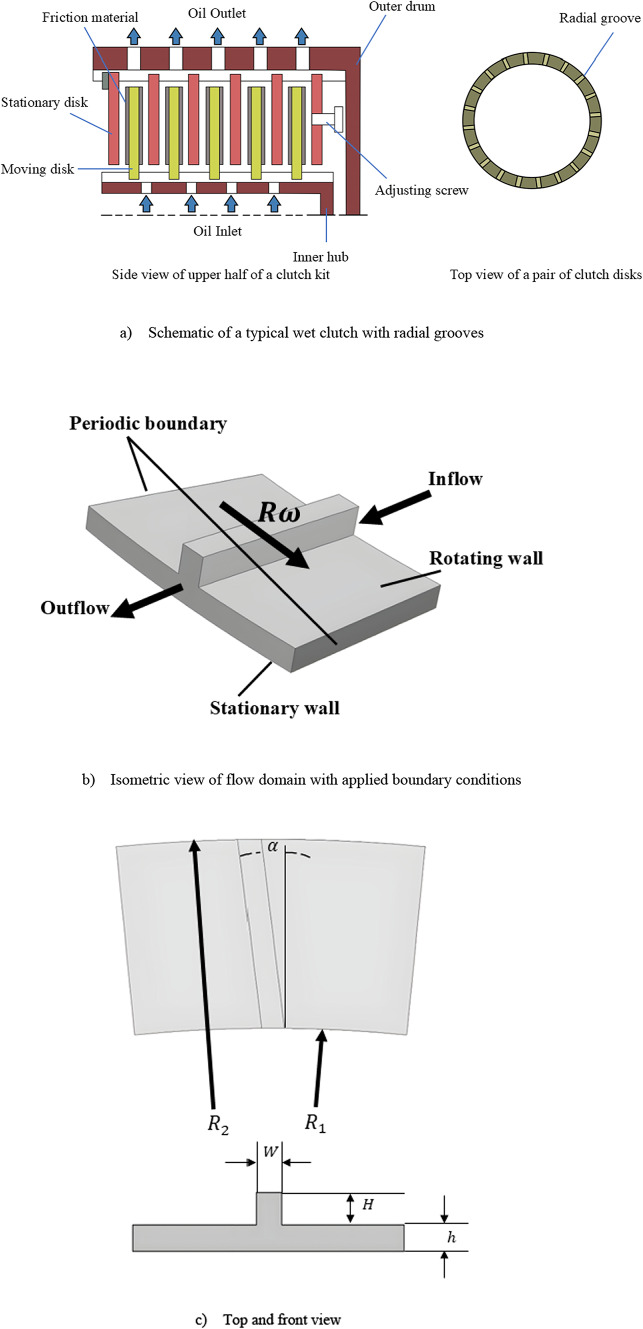
Sketch of the simulation domain.

Figure 1 illustrates the schematic of a typical wet clutch and the geometry used in this study, featuring two parallel disks: one grooved and the other smooth (non-grooved). The key geometric parameters, including the inner radius, ${R}_{1}$, outer radius, ${R}_{2}$, gap clearance, $h$, groove height, $H$, groove width, $W$, and groove angle, $\alpha$, along with their studied ranges, are summarized in Table 1. The reference clutch geometry was adopted from the work of Sadafi et al.^18^. To minimize the computational cost and time, a 1/32 fraction of the full clutch geometry was modeled, with periodic boundary conditions applied to the lateral surfaces. The grooved disk rotates at a specified angular velocity, $\omega$, while the smooth disk remains stationary. Oil is injected radially inward at the inner radius under a constant flow rate, $\dot{m}$, and exits at the outer radius. This setup enables the analysis of flow dynamics and aeration effects within the clutch assembly.

**Table 1:** Geometrical parameters and their ranges.

Parameter	Symbol	Range
Inner radius	*R*_1_	70–85 mm
Outer radius	*R*_2_	90–110 mm
Gap between two disks	*h*	0.2–2 mm
Groove depth	*H*	0.2–2 mm
Groove width	*W*	0.2–2 mm
Groove angle	*α*	0°–20°

## Methodology and theoretical background

### Analytic model

This study utilizes the analytical model originally developed by Leister et al.^14^ and later extended to multiphase conditions by Sadafi et al.^18^. Leister et al.^14^ applied the hydraulic diameter concept to simplify groove-induced equation complexities. The model calculates an equivalent gap for grooved clutches using Eq. (1), which is then substituted into Eq. (2). Equation (2) is used to calculate ${r}_{0}$. The main assumption in the analytical model proposed by Sadafi et al. is that, under two-phase conditions, oil and air are completely separated—oil occupies the region from ${R}_{1}$ to ${r}_{0}$, and air from ${r}_{0}$ to ${R}_{2}$. Although this assumption is not physically accurate, it allows for simpler mathematical operations. Substituting ${r}_{0}$ into Eq. (3) yields the oil volume fraction $\beta$, which is then incorporated into Eq. (4) to calculate the drag torque for the two-phase condition. The critical speed is derived from Eq. (2) by substituting $r={R}_{2}$ and solving for $\omega$.

1 $${h}_{\text{eq}}=\frac{{D}_{h}}{2}=\frac{2A}{p}=\frac{h\pi \left({R}_{1}+{R}_{2}\right)+nHW}{\pi \left({R}_{1}+{R}_{2}\right)+nH}$$

2 $$-\frac{6\mu Q}{\pi {{h}_{eq}}^{3}}Ln\left(\frac{{r}_{0}}{{R}_{1}}\right)+ \frac{3\rho {\omega }^{2}}{20}\left({{r}_{0}}^{2}-{{R}_{1}}^{2}\right)-\frac{2\sigma cos\varphi }{{h}_{eq}}=0$$

3 $$\upbeta =\frac{{r}_{0}^{2}-{R}_{1}^{2}}{{R}_{2}^{2}-{R}_{1}^{2}}$$

4 $$T= \frac{\upbeta \pi \mu \omega }{2{h}_{eq}} \left({{R}_{2}}^{4}-{{R}_{1}}^{4}\right)$$

In the above equations, $T$ represents the drag torque, ${h}_{\text{eq}}$ is the equivalent gap between two disks, $n$ denotes the number of grooves, $Q$ is the volumetric flow rate, $\sigma$ is the surface tension, $\varphi$ is the contact angle, and $\upbeta$ is the oil volume fraction.

In the following sections, the results of the analytical model are compared with numerical data that have been previously validated against experimental results. As will be shown, the analytical results show strong agreement with numerical data in both magnitude and trends. Consequently, the analytical model is employed to generate the training database, enabling machine learning analysis and optimization while minimizing computational expense and runtime. The CPU model used for the simulations is Intel(R) Xeon(R) E5-2695 v3. The number of CPU cores used for the simulations was 22, and each two-phase CFD simulation requires approximately 48 h to reach convergence. Given this high computational cost, employing an analytical approach is a reasonable and efficient alternative for the present problem.

### Data-driven modeling approach

#### Sampling strategy

Database generation constitutes the initial step in machine learning studies. Various algorithms exist for sampling based on the number and the range of features (input variables) and targets (output variable) of the problem. Ensuring a uniform distribution of samples across the design space is essential to avoid bias toward specific feature values, which can negatively affect model accuracy. Several comprehensive studies have evaluated and compared different sampling strategies in the literature^25–27^. Among the most widely used methods are Monte Carlo sampling (MCS), Latin hypercube sampling (LHS), and Sobol sequences^28^. The Sobol method generates samples using a deterministic low-discrepancy sequence that ensures rapid and uniform convergence across the input space. This technique has shown strong performance in data-driven modeling, sensitivity analysis, and model calibration tasks^27^. A detailed explanation of the Sobol algorithm can be found in Sobol^29^.

This study employed Sobol sequence sampling across seven input parameters: six geometric features, as previously described: ${R}_{1},{R}_{2},H,h,W,\alpha$ and one fluid property: $\mu$ (dynamic viscosity). The output parameter, drag torque, was calculated using the analytical model developed by Sadafi et al.^18^. Two databases with 600 samples have been generated for data-driven modeling, one containing only geometric parameters and the second incorporating dynamic viscosity to improve the model accuracy. The drag torque (output parameter) was calculated for each sample over a rotational speed range of 0–120 rad/s, with increments of 10 rad/s. The model was trained on two other databases with 3000 and 6000 samples. The mean squared error (MSE) values for these models were 1.22 × 10^−4^ and 8.37 × 10^−5^, while the R^2^ values were 0.99908 and 0.9998, respectively. As will be demonstrated in subsequent sections, the metric values belonged to the case with 3000 samples show no significant difference compared to those obtained for the model trained on the 600-sample database. Although the MSE value decreased significantly for the case with 6000 samples, this was accompanied by a large increase in runtime and computational costs. Furthermore, an excessive increase in the number of samples could potentially lead to overfitting. Therefore, to prevent overfitting and reduce computational costs, the database with 600 samples was utilized. In addition, it will be demonstrated that the optimization results correspond closely with the CFD analyses, thereby confirming the accuracy of the predictions.

#### Artificial neural network

**Fig. 2:**
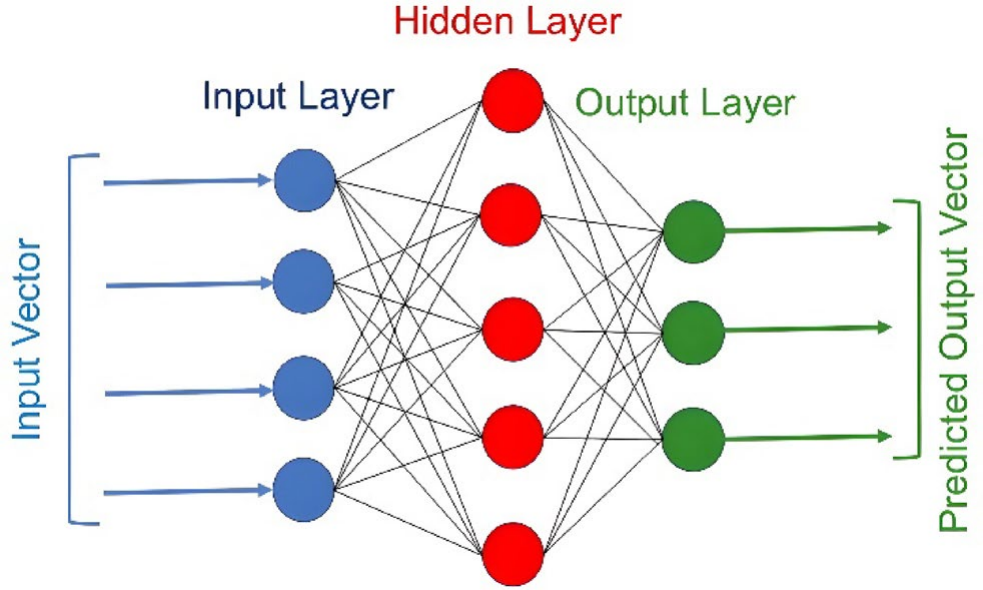
Schematic of an artificial neural network.

Machine learning algorithms have gained widespread popularity for identifying complex input–output relationships across scientific disciplines. Artificial neural networks represent a widely-used data-driven approach inspired by biological neural systems. As illustrated in Fig. 2, the basic ANN structure consists of: (1) an input layer, (2) one or more hidden layers, and (3) an output layer. Each layer contains processing units (neurons) characterized by specific activation functions that govern input–output relationships. Information flows unidirectionally (feed-forward) unless recurrent—or feedback—connections incorporate predicted outputs as subsequent inputs. Both feed-forward and feedback architectures can model nonlinear relationships^30^.

The network architecture (number of hidden layers and neurons per layer) is problem-dependent. Neurons interconnect via adjustable weight coefficients. The computational process propagates sequentially from the input vector through hidden layers to the output layer, applying nonlinear transformations at each stage to convert inputs to outputs. A key advantage of artificial neural networks lies in their ability to efficiently model coupled nonlinear input–output relationships. This capability, however, necessitates a learning algorithm that systematically adjusts inter-nodal weight coefficients to ensure accurate mapping between input and output vectors. The backpropagation algorithm stands as the most widely adopted learning method for artificial neural networks. In this process, input vectors first propagate forward through the network to produce output predictions. The discrepancy between these predictions and target values is then quantified as an error metric. This error signal propagates backward through the network’s layers and a gradient descent method adjusts the weight coefficients to reduce prediction errors. This process repeats iteratively through sample data and the weight adjustments progressively refine the network’s ability to approximate complex nonlinear relationships between inputs and outputs^31,32^.

The ANN training was implemented in MATLAB R2023A using Levenberg–Marquardt backpropagation. The selection of the Levenberg–Marquardt algorithm for training the artificial neural network was based on its proven efficiency in optimizing medium-sized networks and its robustness in handling nonlinear relationships. The LM algorithm combines the advantages of gradient descent and Gauss–Newton methods, ensuring rapid convergence and stability, which is particularly beneficial for complex datasets^32^. The network structure employed three hidden layers, with mean square error (MSE), mean absolute error (MAE), mean absolute percentage error (MAPE), and R^2^ serving as performance metrics. The number of neurons per each hidden layer are 64, 16, and 8, respectively. 70% of the sample data was used for training, 15% for test, and 15% for validation. The mathematical definitions of MSE and R^2^ are shown in Eqs. (5) and (6), respectively.

5 $$MSE=\frac{1}{N}\sum_{i=1}^{N}\left({C}_{{p}_{est}}-{C}_{{p}_{tar}}\right)^{2}$$

6 $$R^{2} = \frac{{\mathop \sum \nolimits_{{i = 1}}^{N} \left( {C_{{p_{est}}} - C_{{p_{avg}}} } \right)^{2} }}{{\mathop \sum \nolimits_{{i = 1}}^{N} \left( {C_{{p_{tar}}} - C_{{p_{avg}}} } \right)^{2} }}$$

where N is the number of samples, ${{C}_{{p}_{tar}}}$ is the target value, ${{C}_{{p}_{est}}}$ is the estimated value and ${{C}_{{p}_{avg}}}$ is the arithmetic average value.

### Optimization methodology

Substantial research efforts have addressed complex engineering optimization challenges to reach a better performance of efficiency. An optimization problem involves determining parameter values that maximize or minimize an objective function, considering some specified limitations. Thereby, there are three key parameters influencing the result of the optimization process: (1) the objective function, (2) the decision variables influencing this function’s value, and (3) the constraints that must be satisfied^33^. This study focuses on optimizing radial groove geometry in clutches to minimize the drag torque value. The genetic algorithm was selected as the optimization methodology.

The genetic algorithms comprise stochastic optimization methods inspired by biological evolution and natural selection principles. Natural selection describes the evolutionary process whereby organisms with advantageous traits achieve greater reproductive success, causing these traits to become predominant in subsequent generations. The computational framework mimics natural selection through the selection, crossover, and mutation processes^34^.

This study implements the genetic algorithm through MATLAB R2023A GA function, utilizing the previously developed ANN as the objective function. The optimization process is single-objective. The aim is to reduce the drag torque, which inherently leads to a reduction in the critical speed. The defined ranges of the design variables are identical to the range specified for the neural network input variables (Table 1).

### Numerical simulation methodology

#### Governing equations

The governing equations for fluid flow problems are the well-known continuity and the momentum equations, as outlined below:

7 $$\frac{\partial {u}_{i}}{\partial {x}_{i}}=0$$

8 $$\rho \left(\frac{\partial {u}_{i}}{\partial t}+{u}_{j}\frac{\partial {u}_{i}}{\partial {x}_{j}}\right)=-\frac{\partial p}{\partial {x}_{i}}+\mu \frac{{\partial }^{2}{u}_{i}}{\partial {x}_{j}\partial {x}_{j}}+{f}_{i}$$

where ${u}_{i}$ represent velocity component in the i-th direction, ${x}_{i}$ is the spatial coordinate in the i-th direction, $p$ is the pressure, $t$ is the time, $\mu$ denotes the dynamic viscosity, and ${f}_{i}$ is the body force per unit volume in i-th direction.

Once flow becomes two-phase, the above equations are not adequate to describe the heterogeneous structure of the two immiscible fluids. Previous studies have demonstrated that the distribution of two-phase flow is highly non-uniform and characterized by the presence of separate air bubbles^11^. To enable the analysis of the two-phase flow, the volume of fluid (VOF) approach is employed. This approach is highly recommended in prior researches for its effectiveness and accuracy in capturing the interface between different fluids^1,9,24^.

In this model, a volume fraction $\beta$ is introduced to indicate whether oil occupies a larger portion of a computational cell relative to the total cell volume. The interface between the phases is defined by values of $0<\beta <1$. Additionally, the sum of the volume fractions within each cell must equal one, as expressed below:

9 $${\beta }_{oil}+{\beta }_{air}=1$$

The density and viscosity terms in these equations are expressed as functions of the volume fractions for both phases, as defined below:

10 $$\rho ={\beta }_{oil}{\rho }_{oil}+{\beta }_{air}{\rho }_{air}$$

11 $$\mu ={\beta }_{oil}{\mu }_{oil}+{\beta }_{air}{\mu }_{air}$$

The VOF model employs a shared set of governing equations, solving a single momentum equation and continuity equation for all phases. This approach yields a velocity field that intrinsically couples the phases at their interface. For precise interface tracking, an additional transport equation is solved for the volume fraction $\beta$ of one phase:

12 $$\frac{{\partial \left( {\beta _{{oil}} } \right)}}{{\partial t}} + \frac{{\partial \left( {\beta _{{oil}} u_{i} } \right)}}{{\partial x_{i} }} = 0$$

#### CFD solver and boundary conditions

The numerical simulations were performed using the commercial software ANSYS Fluent 2023 R2 for both the single-phase and two-phase flow analyses. For single-phase simulations, the pressure–velocity coupling was applied using the SIMPLE scheme, while for the two-phase flows, the coupled scheme was employed to enhance numerical stability*.* As established in the authors’ previous study^18^, the typical cases in this application used laminar flow regime; therefore, the flow regime in the present work is also modeled as laminar. The computational domain employed the following boundary conditions: (1) a fixed mass flow rate at the inner radius inlet, (2) a pressure outlet with ${p}_{gauge}=0$ at the outlet boundary, and (3) rotational periodic conditions for the side boundaries. The grooved disk was modeled with a moving wall boundary condition at constant rotational speed, while the smooth disk was treated as a stationary no-slip wall. Due to the geometric complexity introduced by the grooves—particularly their sidewalls being perpendicular to the rotation direction—direct application of rotational velocity to the grooved disk proved problematic. To resolve this, the reference frame was set to rotate at the target speed while imposing counter-rotation on the smooth disk, thereby achieving the desired relative motion. The complete boundary condition configuration was illustrated in Fig. 1.

For multiphase simulations, the inlet boundary condition specifies both the oil mass flow rate and volume fraction, with ${\upbeta }_{\text{oil}}= 1 \left({\upbeta }_{\text{air}} = 0\right)$ to represent pure oil injection from the inner radius. The outlet boundary was configured with a backflow volume fraction of ${\upbeta }_{\text{oil}} = 0$, preventing oil return into the computational domain. This boundary condition allows for aeration at high rotational speeds.

The oil mass for rate is set to 3 L/min. The Reynolds number ranged from 6.32 to 190.82 for air and from 7.91 to 224.86 for oil across different cases. The contact angle was set to 0°.

#### Mesh generation

A structured hexahedral mesh was generated for each configuration by the commercial ANSYS Meshing software. While the total cell count varied with geometric dimensions, the average of 350,000 cells exists in each mesh. To resolve the boundary layer effects, inflation layers were implemented along the disk edges with a minimum thickness of 2 × 10^−5^ m, ensuring accurate capture of near-wall gradients. A grid independence study was conducted by evaluating three mesh resolutions for a representative case with 2.0 × 10^5^, 3.4 × 10^5^, and 1.5 × 10^6^ cells, respectively. The corresponding drag torque was 0.2614, 0.2622, and 0.2628 N m, respectively. The difference between second and third case is approximately 0.2%, demonstrating negligible dependence on grid size.

#### Time-stepping and convergence criteria

For single-phase cases, a steady-state simulation was conducted. In two-phase cases, the pseudo-transient approach was adopted to address two key challenges: (1) the prohibitive computational cost of fully transient simulations, and (2) the extended convergence time typically required for such configurations. The pseudo-transient formulation offers significant advantages, including enhanced numerical stability, accelerated convergence rates, and reduced computational expense^35^. This approach is particularly appropriate for the current study, as the flow system evolves toward a stationary state where transient effects become negligible. The “automatic” time-stepping method was employed in the simulations to balance accuracy and computational efficiency. A time scale factor of 0.8 was selected to enhance numerical stability while maintaining sufficiently small time steps for accurate interface tracking. In addition, the length scale method was set to “conservative” to ensure robust convergence in the presence of strong gradients and multiphase interactions.

The convergence criterion is met when the L2-norm residuals fall below 5 × 10^−5^ for single-phase flows and 10^−5^ for multiphase flows across all governing equations. Even though simulations were continued until the drag torque on the stationary disk and the average of oil volume fraction in the whole domain reached a constant value in terms of time.

## Results and discussion

This section begins with the validation of the analytical model against numerical results and proceeds with the generation of a dataset using Sobol sampling. Based on this data, an artificial neural network model is developed to capture the underlying relationships. The trained ANN is then used within a genetic algorithm framework to perform optimization, and the optimized results are subsequently verified through numerical simulations. Finally, the impact of geometric variations resulting from the optimization is discussed in detail using CFD analysis.

### CFD model validation

**Fig. 3:**
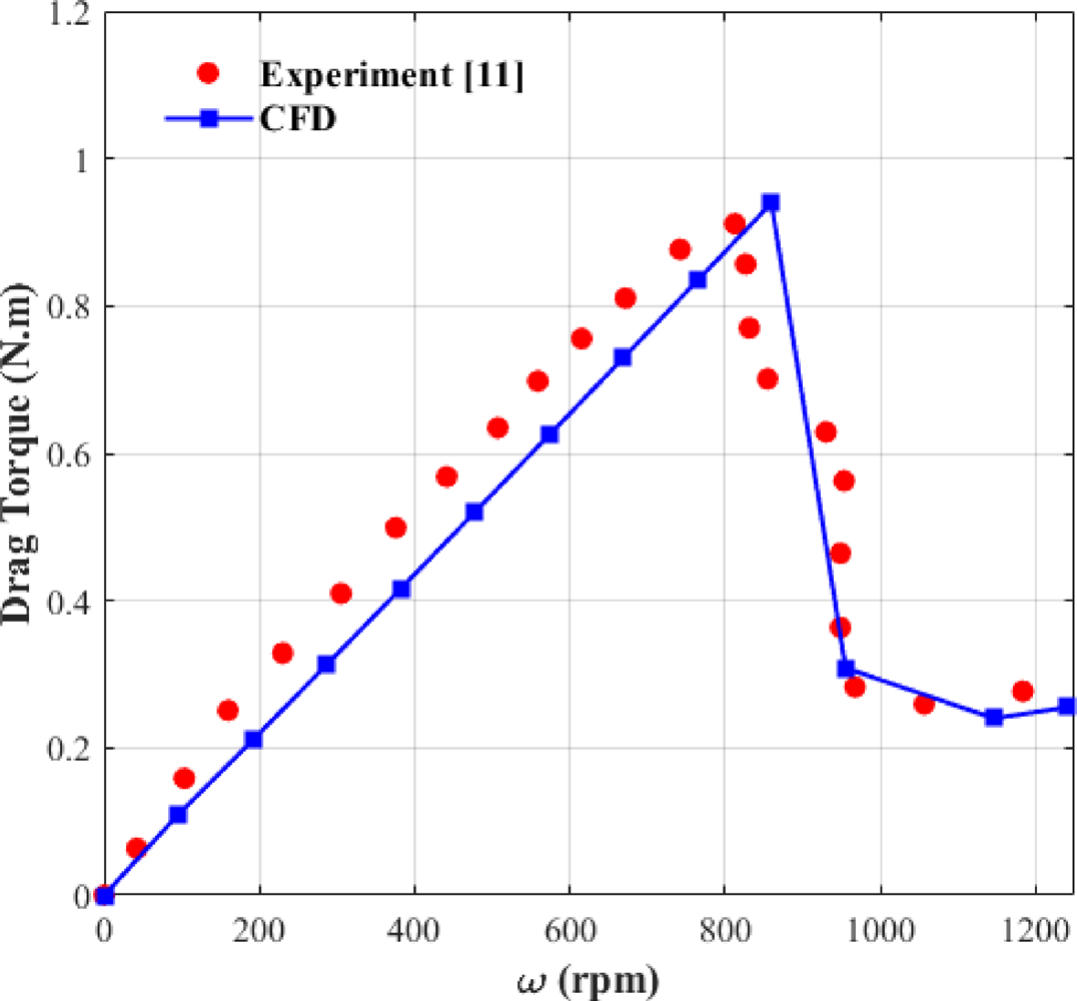
Comparison of CFD and experiment^11^ for the validation of CFD model.

The CFD setup of this study was implemented to simulate one of the cases studied in Ref.^11^ to assess the accuracy of the numerical results through comparison with experimental data. The related geometric parameters are specified in Table 2. Figure 3 shows the comparison of the drag torque predictions with experimental data, showing strong agreement with a maximum deviation of 4%. The discrepancies occasionally observed between numerical and experimental results may arise from numerical errors and the limitations of the simulations. Nevertheless, such errors will not propagate into the optimization results, as it will be demonstrated that the optimization outcomes exhibit excellent agreement with the numerical results. The close agreement between the CFD and experimental results confirms the reliability of the numerical approach for subsequent analyses.

**Table 2:** Geometrical parameters of the validation case studied in Ref.^11^.

Parameter	Symbol	Value (mm)
Inner radius	*R*_1_	67.5
Outer radius	*R*_2_	84.25
Gap between two disks	*h*	0.15
Groove depth	*H*	0.6
Groove width	*W*	1.5

### Analytic model performance

Based on standard sampling practices for data-driven models, constructing a training set with seven input variables typically requires approximately 500–1000 distinct numerical setups. Extending the analysis to multiple rotational speeds increases the total number of required CFD simulations to approximately ten times the size of the initial database. This substantial computational demand highlights the need for more efficient alternatives in database generation for optimization studies.

The analytical model proposed by Leister et al.^14^, which was extended to multiphase conditions by the authors previous work^18^ shows reasonable agreement with numerical data for drag torque prediction. However, comprehensive evaluation of this model’s accuracy across different geometric parameters remains unaddressed in the previous study. This section compares the analytical model’s predictions with numerical results obtained for various geometric parameters to assess the trend and magnitude captured by the analytical model. This comparison confirms the model’s accuracy for subsequent clutch geometry optimization.

**Fig. 4:**
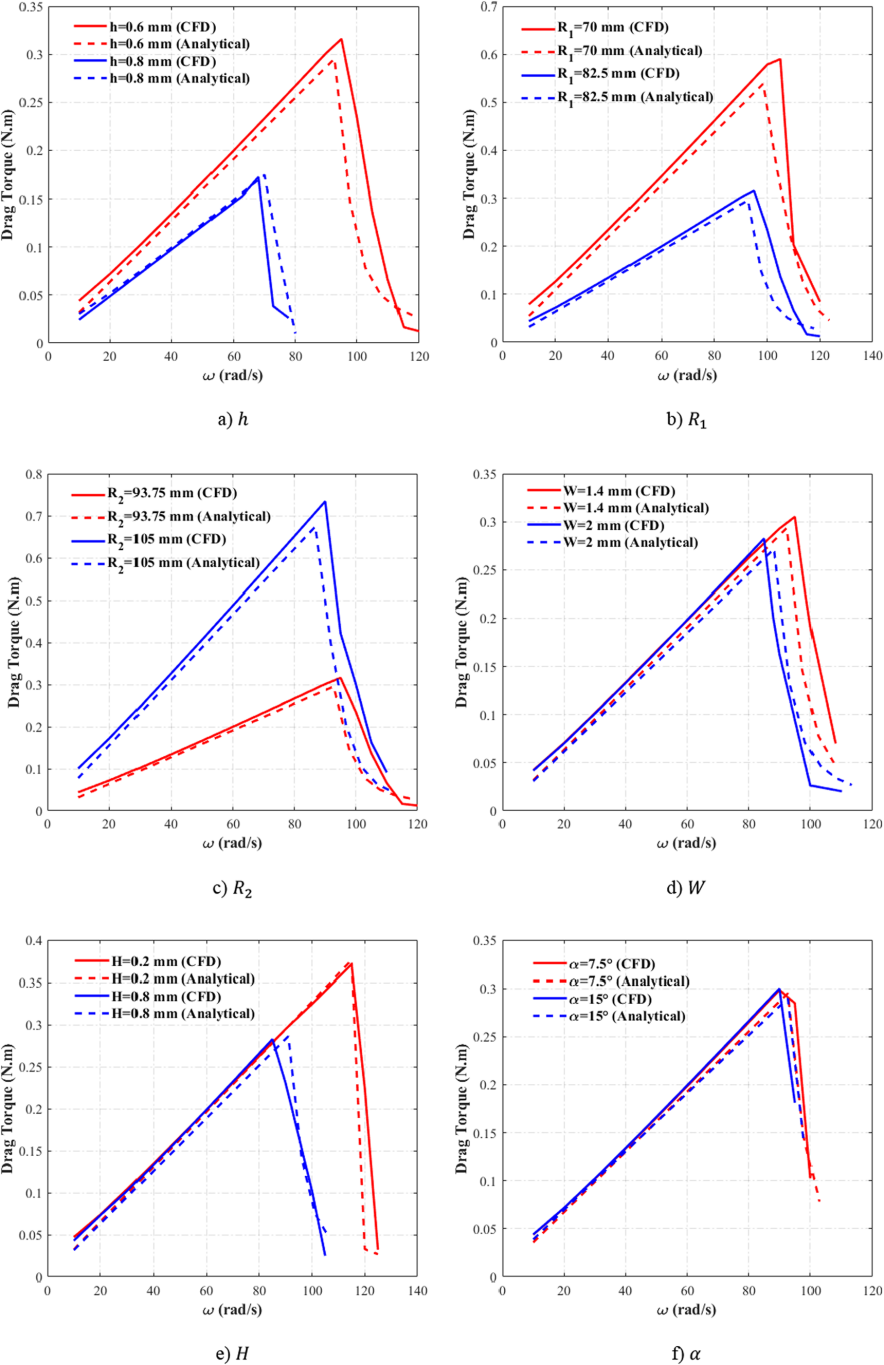
The compassion of CFD and analytical results^18^ for various geometric parameters.

Figure 4a compares the drag torque predictions between the analytical model and CFD for *h* = 0.6 mm and *h* = 0.8 mm, while the other geometric parameters remain constant. The analytical model accurately captures the trend of drag torque reduction with increasing groove depth, although minor deviations are observed. The model underestimates critical speed by 3% and shows a 6% discrepancy in the peak drag torque compared to the CFD results. These differences primarily arise from critical speed measurement errors, leading to a slight mismatch between the curves.

Figure 4b represents this comparison for various ${R}_{1}$ values, demonstrating consistent agreement in both the drag torque trends and magnitudes. The analytical results maintain a maximum of 8% deviation from CFD data for the drag torque, while critical speed predictions for *R*_1_ = 70 mm show a 6% difference relative to numerical values. Figure 4c extends this analysis to varying *R*_2_ values, with the analytical model again matching the numerical results trend and magnitude. The maximum discrepancy occurs at *R*_2_ = 105 mm, where the peak drag torque values differ by approximately 8% between the analytical and CFD results.

Figures 4d and 4e present the comparisons for different $W$ and $H$, while the other parameters remain constant. Figure 4d confirms that the analytical model captures the drag torque reduction trend with increasing $W$. However, a significant 37% discrepancy emerges at *ω* = 98 rad/s. This error originates from Sadafi et al.’s^18^ basic assumption, which was modeling multiphase flow as concentric annular regions (inner oil ring, outer air ring)—a physically inaccurate but mathematically convenient simplification. While this assumption may create initial mismatches at multiphase transition speeds, the results converge to reasonable accuracy at higher rotational velocities^18^. Figure 4e demonstrates strong agreement between analytical and CFD results, with the maximum discrepancy (approximately 5%) occurring at the critical speed for the *H* = 0.8 mm case.

Figure 4f shows the comparison of the analytical and CFD results for different groove angle. The numerical data indicate that the effect of groove angle on the drag torque is not considerable. This behavior is also confirmed in the analytical results and the values change with groove angle very slightly. Nevertheless, the analytical results maintain agreement with CFD data, with a maximum observed deviation of 6%.

In summary, the analytical model developed by Sadafi et al.^18^ demonstrates reliable prediction of both the drag torque and critical speed trends and magnitudes. This comparison with pre-validated numerical results confirms the model’s accuracy for generating the required database for the optimization process. While localized discrepancies occur at limited data points, these do not substantially compromise the model’s overall accuracy. It should be noted that the primary purpose of this figure is to demonstrate that the analytical model is capable of capturing the influence of all relevant parameters on drag torque, thereby providing reliable physical trends to guide the optimization process. These deviations may introduce a degree of error into the optimization results; however, this limitation will be addressed by validating the optimized designs against numerical simulations.

### ANN training performance

As mentioned in methodology, two distinct databases were developed for machine learning training. The first database incorporates six geometric input parameters (*R*_1_, *R*_2_, *H*, *h*, *W*, *α*) within their specified ranges and drag torque as the output, while the second database additionally includes dynamic viscosity (*μ*) as an input parameter to enhance training accuracy. These datasets form the basis for training two artificial neural networks, designated as “Model 1” and “Model 2”, respectively.

**Fig. 5:**
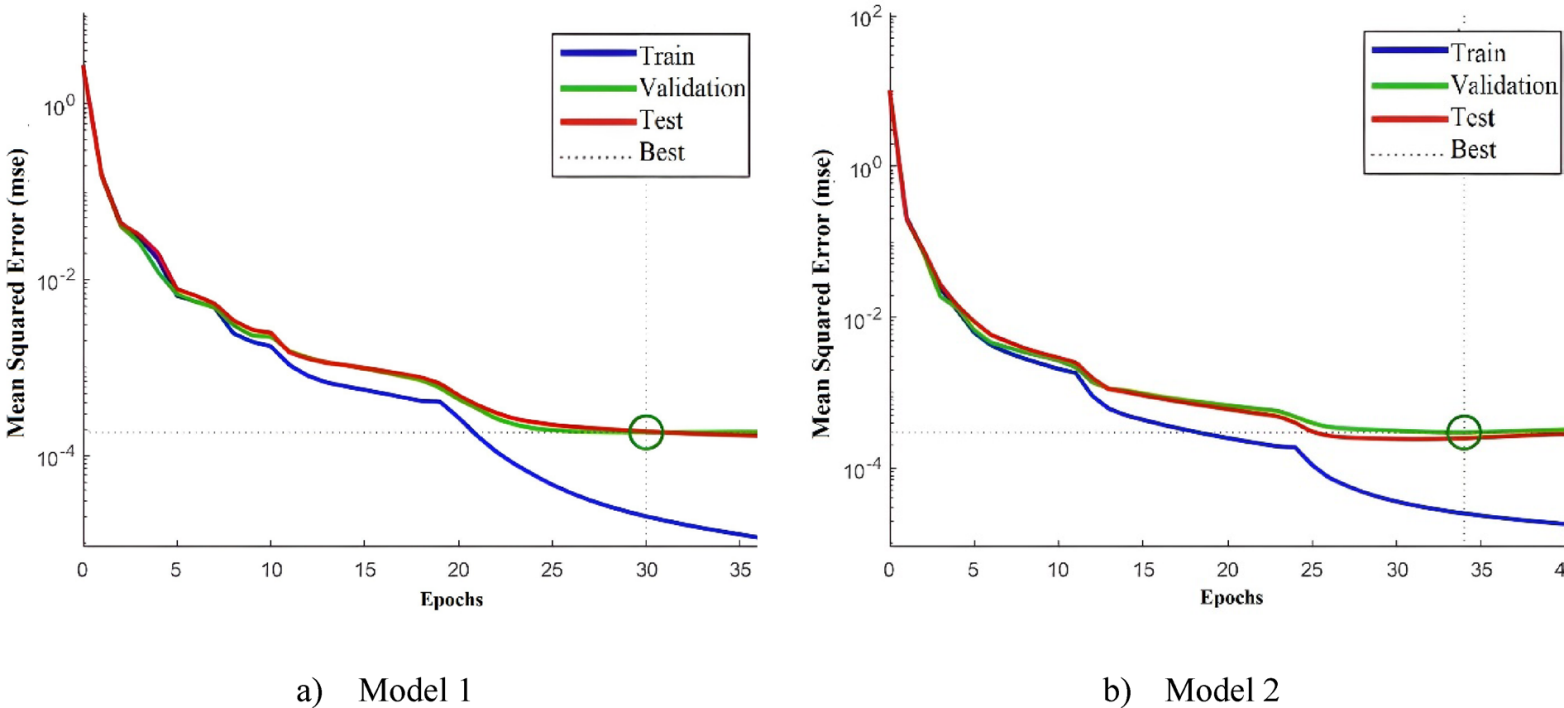
MSE convergence history of the trained models.

Table 3 presents the performance metrics for both models across training, testing, and validation phases. In regression analysis, optimal performance is indicated by R^2^ values close to 1 and MSE and MAE values approaching zero, while lower MAPE values are preferred since they are expressed as percentages. The results confirm strong model accuracy, with both models achieving R^2^ values near unity, MSE on the order of 10^−4^, and MAE on the order of 10⁻^3^. Moreover, the MAPE values are approximately 5% for Model 1 and 9% for Model 2, both of which are reasonable. These findings indicate that Model 1 yields slightly lower prediction errors compared to Model 2. Figure 5 illustrates the MSE convergence history for both Model 1 and Model 2.

**Table 3:** Accuracy metric value of the trained ANNs.

Metric	Model 1			Model 2		
	Train	Test	Validation	Train	Test	Validation
R^2^	0.99987	0.99890	0.99872	0.99980	0.99822	0.99716
MSE	2.02 × 10^−5^	1.86 × 10^−4^	1.82 × 10^−4^	2.52 × 10^−5^	2.51 × 10^−4^	2.98 × 10^−4^
MAE	0.002	0.0025	0.0036	0.0037	0.0063	0.0066
MAPE	4.7578	5.6245	5.8896	7.4691	9.5409	9.3980

**Fig. 6:**
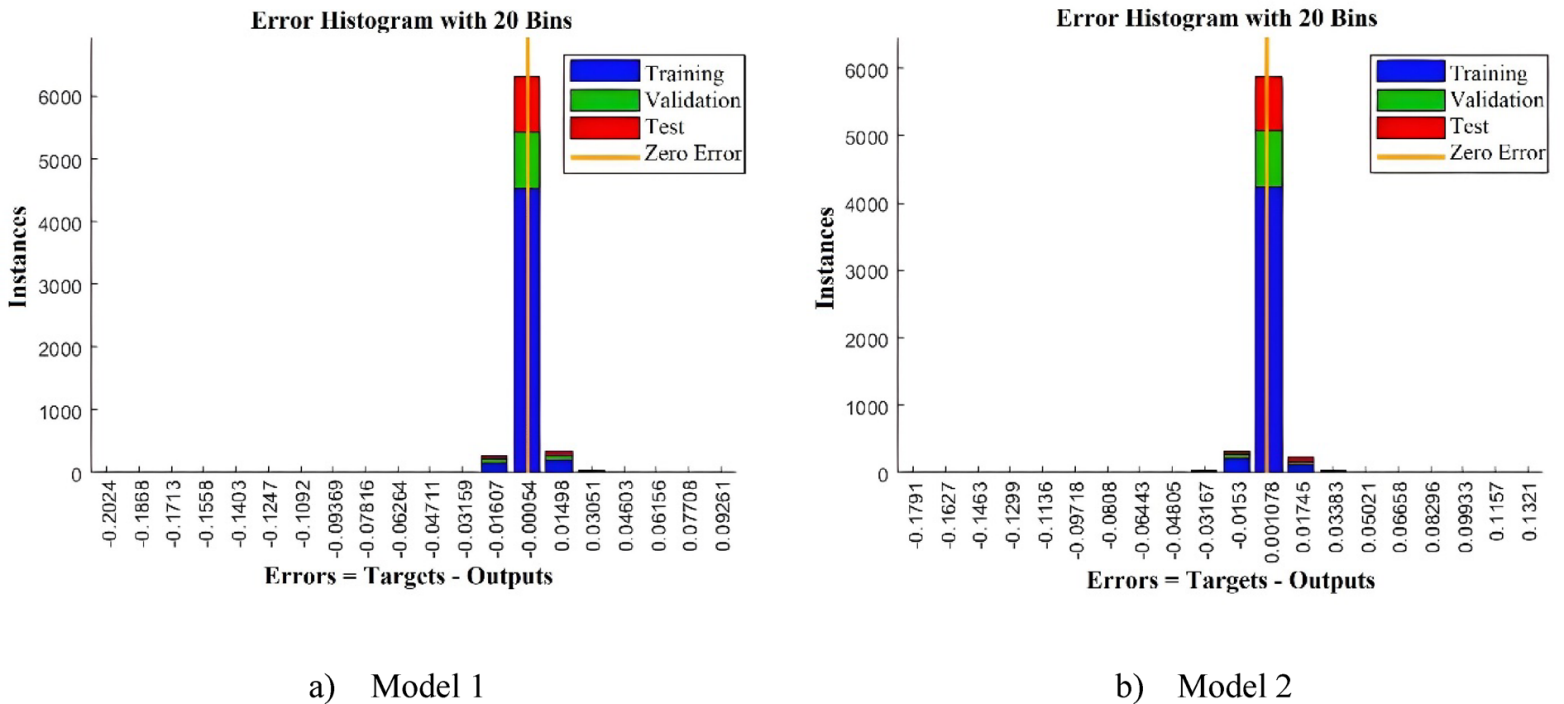
Histogram distribution of the trained models.

**Fig. 7:**
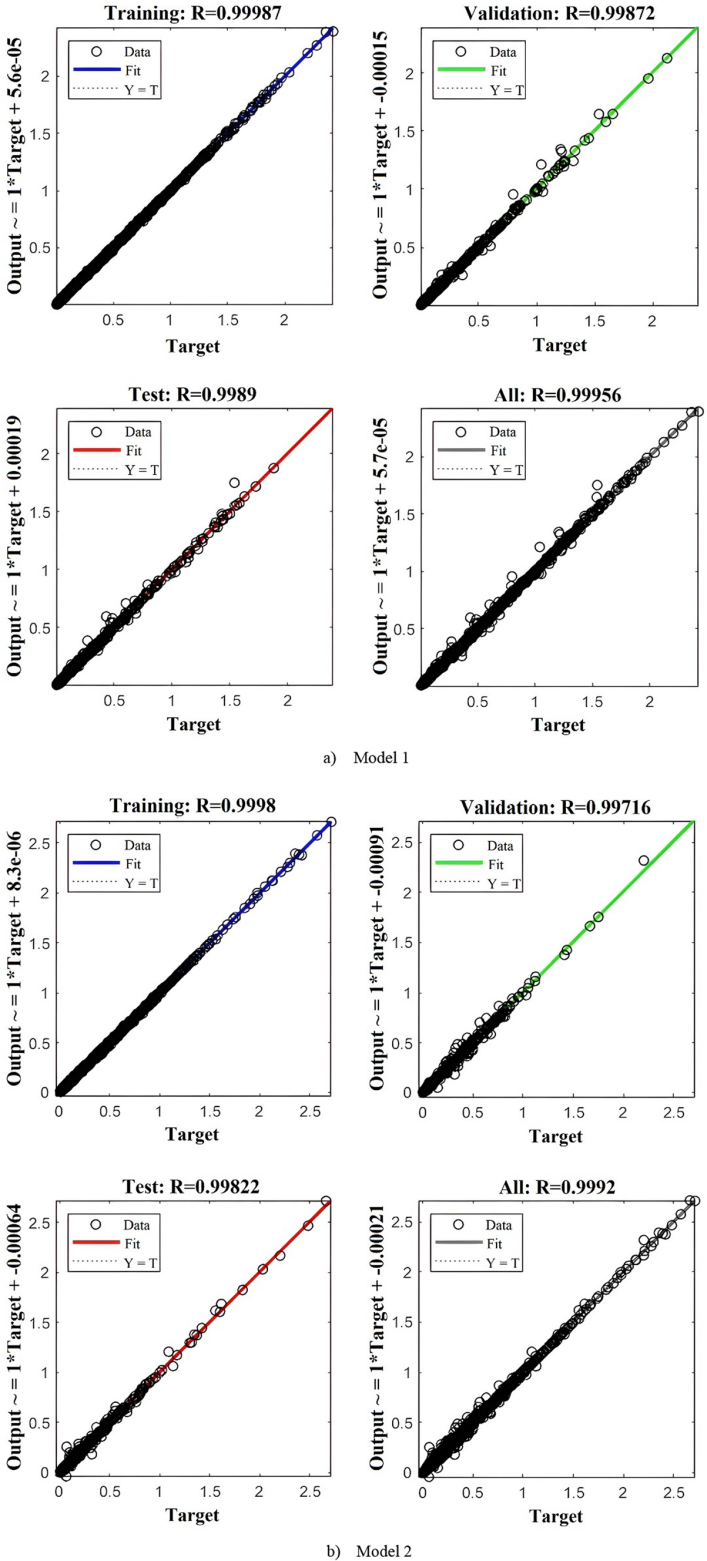
Predicted versus actual plots of the trained models.

The histogram distributions for both models in Fig. 6 exhibit strong symmetry, indicating balanced prediction behavior without over- or under-estimation. This conclusion is further supported by predicted vs. actual plots in Fig. 7, demonstrating high model accuracy while revealing no discernible systematic trends or outlier clusters.

### Optimization results

Two artificial neural networks developed in the previous section were implemented as the objective function for the optimization process. The optimization was performed using the genetic algorithm (GA) function in MATLAB, with a population size of 100 and a maximum of 700 generations. It should be noted that the optimization was conducted exclusively on the geometric variables and the dynamic viscosity ($\mu$), within a range of rotational speeds (0–120 rad/s), while the mass flow rate was assumed to be constant. Four distinct cases, designated as Cases 1–4, were examined. Cases 1 and 2 were optimized using Model 1, which considers only geometric inputs, whereas Cases 3 and 4 were optimized using Model 2, which incorporates $\mu$ as an additional input.

The major difference between Cases 1 and 2 lies in the constraint applied to the variation of ${R}_{1}$ in Case 2. In practical applications, the inner radius of the shaft is typically close to the shaft radius, and modifying the gearbox shaft diameter to optimize an existing clutch is often infeasible. A similar difference exists between Cases 3 and 4. Further details of these cases are provided in Table 4. The dynamic viscosity was also held constant across all cases to ensure comparability with numerical data. Overall, Cases 3 and 4 are expected to yield more realistic results, since they incorporate a broader set of input variables and apply more practical constraints in the optimization process.

**Table 4:** Details of the optimization cases.

Case	Objective function	Input Variables	Remark
1	Model 1	*R*_1_, *R*_2_, *H*, *h*, *W*, *α*	–
2	Model 1	*R*_1_, *R*_2_, *H*, *h*, *W*, *α*	*R*_1_ is held constant
3	Model 2	*R*_1_, *R*_2_, *H*, *h*, *W*, *α*, *μ*	*μ* is held constant
4	Model 2	*R*_1_, *R*_2_, *H*, *h*, *W*, *α*, *μ*	*R*_1_ and *μ* are held constant

**Fig. 8:**
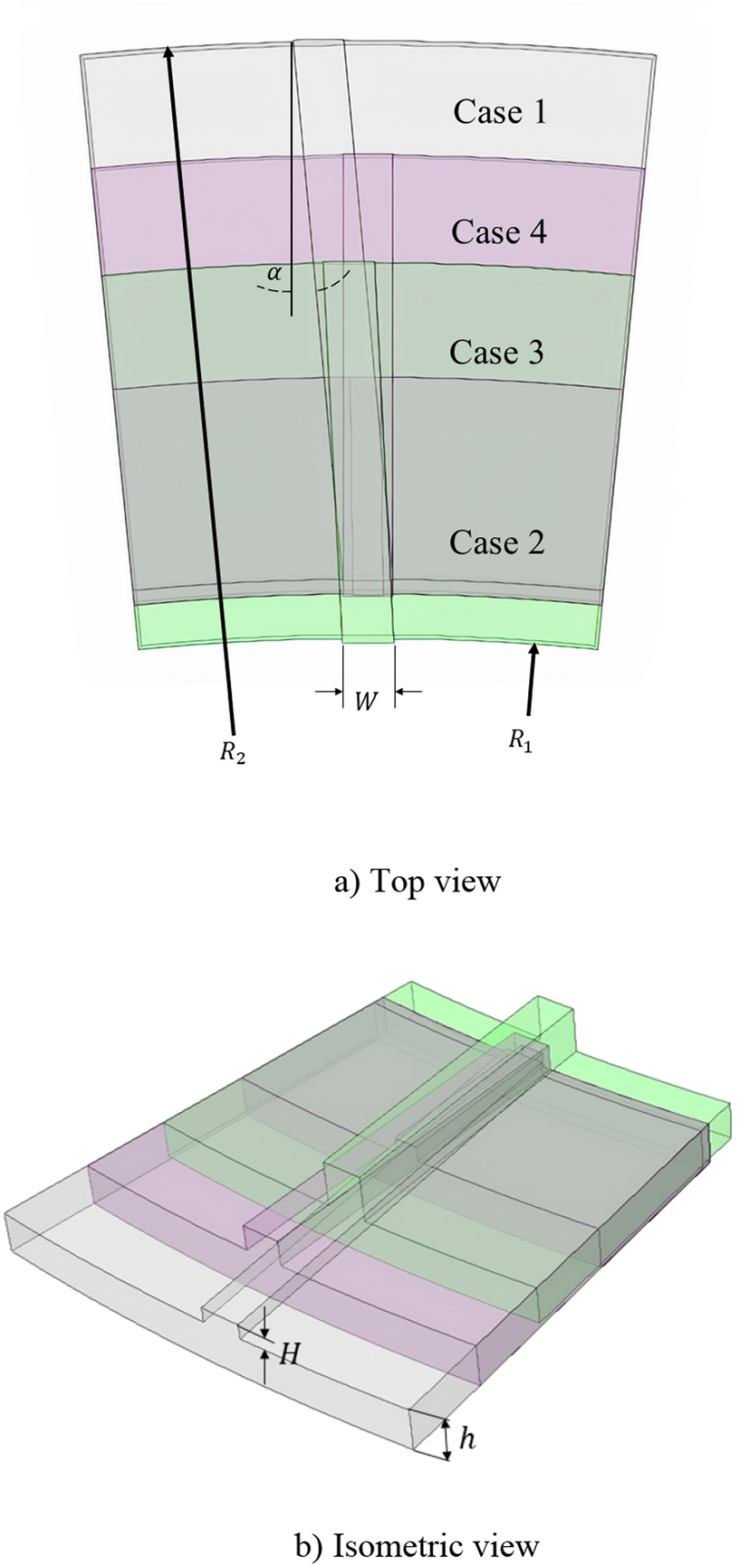
Schematic of the optimized cases.

**Fig. 9:**
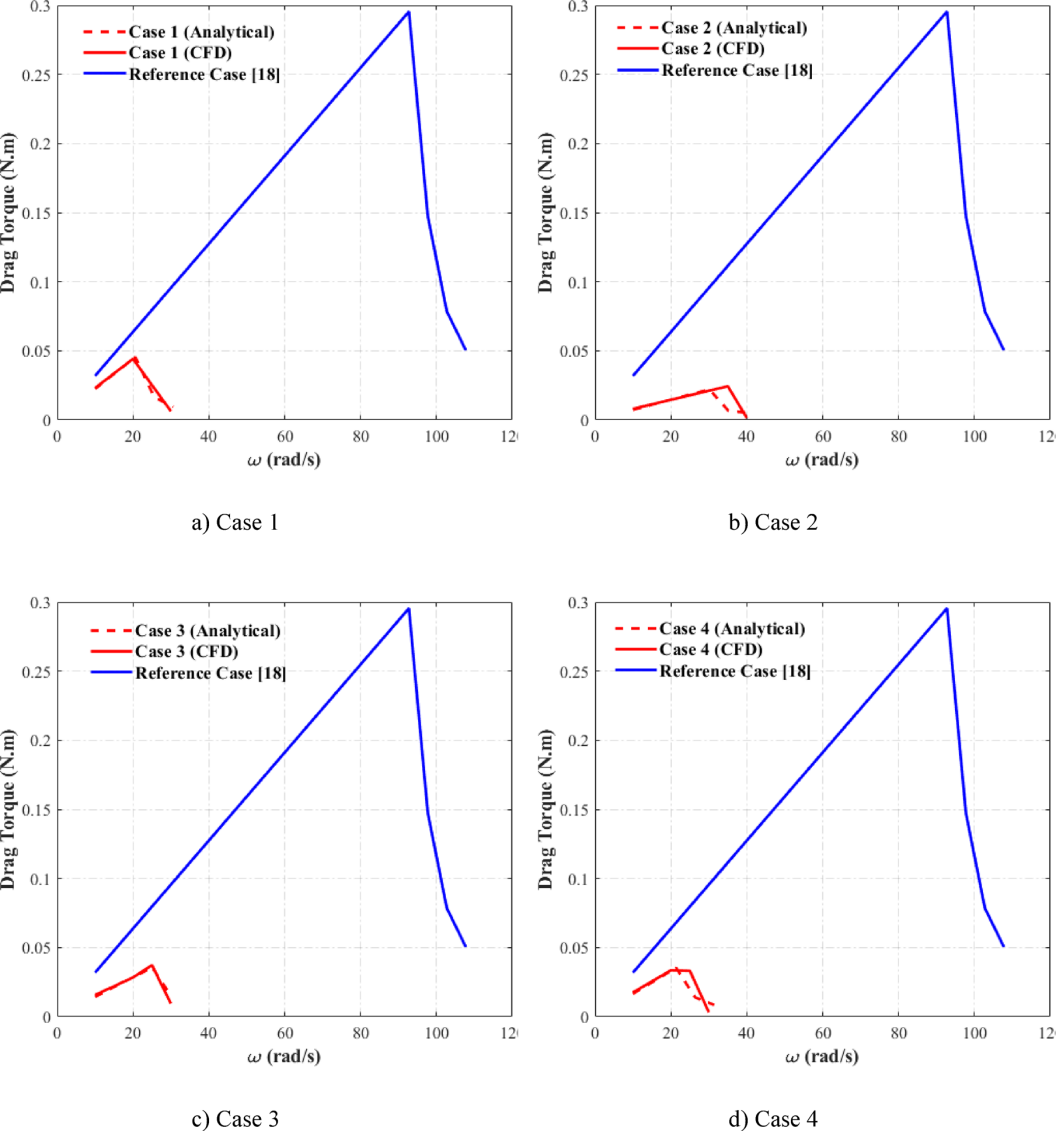
The compassion of CFD and analytical results for optimized cases.

Figure 8 presents a three-dimensional schematic representation of the optimized configurations, in a way that they are comparable for which the values of geometric variables are listed in Table 5. To validate the optimization outcomes, fluid flow simulations were conducted for all cases. Figure 9 displays the drag torque-speed characteristics for all cases, comparing numerical and analytical results. The corresponding curve for the reference case^18^ is included to assess the performance of optimized cases. A reasonable agreement is observed between numerical and analytical results across all cases. A minor discrepancy exists between the two approaches for Case 2, with the CFD-predicted critical speed approximately 5 rad/s higher than the analytical model’s prediction. This deviation remains within acceptable limits. Comparative analysis between the optimized and reference cases reveals the most significant findings. The optimization process yields substantial reductions in both the drag torque and critical speed, as evidenced by the optimized curves lying consistently below those of the reference case. Case 1 demonstrates a substantial reduction in critical speed from 92 to 20 rad/s, representing a 78% decrease. The peak drag torque shows an approximately 85% reduction. This trend persists across all cases: Case 2 exhibits a 66% reduction in the critical speed and 90% reduction in the maximum drag torque, Case 3 shows a 73% decrease in the critical speed with an 88% reduction in the maximum drag torque, and Case 4 achieves a 73% reduction in the critical speed and 89% reduction in the maximum drag torque. Furthermore, it can be concluded that the lower limit for the critical speed in this design is approximately 20 rad/s. These results demonstrate the effectiveness of the optimization process, with the obtained geometries exhibiting superior performance in the drag torque reduction. Besides, the results indicate that the optimization effectively accelerates the onset of aeration and reduces the critical velocity, which corresponds to a lower peak in drag torque and consequently decreases energy loss. Consequently, modifying the reference design according to the optimized configurations would yield significant performance improvements.

**Table 5:** Values of the optimized geometric parameters.

Parameter	Case 1	Case 2	Case 3	Case 4
*H*	0.626 mm	0.62 mm	1.758 mm	1.095 mm
*h*	1.735 mm	1.717 mm	1.555 mm	1.745 mm
*W*	1.742 mm	1.039 mm	1.945 mm	1.69 mm
*α*	5.22°	0°	2.886°	0.043°
*R*_1_	83.01 mm	82.5 mm	80.96 mm	82.5 mm
*R*_2_	101.65 mm	90.01 mm	93.97 mm	97.7 mm

While the optimization results clearly demonstrate the potential for significant drag torque reduction, the manufacturability and practical implementation of the optimized geometries must also be considered. Some of the optimized cases (e.g., Case 4 with a very small groove angle of 0.043° or a relatively large inter-disk gap of ~ 1.75 mm) represent theoretical optima that may not be directly achievable in industrial production. In practice, groove angles below ~ 1° are difficult to machine with consistency, and excessive inter-disk clearances can compromise clutch compactness and oil distribution.

Moreover, changes in geometry can influence secondary performance metrics. For instance, Neupert and Bartel^12^ have shown that groove geometry affects axial forces arising from hydrodynamic pressure distributions. Pan et al.^36^ also demonstrated that the drag torque may increase again at high rotational speeds due to growth of axial forces. Although axial force was not directly included as a constraint in the present optimization, excessively deep grooves or large clearances could alter the pressure field and increase the mechanical load on supporting structures. Similarly, larger gaps may facilitate earlier aeration and reduce drag torque, but they also risk they also may affect the performance under engaged conditions.

Therefore, the optimized geometries highlight valuable design trends—such as the strong influence of gap clearance and groove depth, and the limited effect of groove angle—but their direct implementation requires balancing drag torque reduction against manufacturability, structural loads, thermal management, and durability.

### Effect of geometry on drag torque

Based on the optimization results presented in the previous section, it is evident that nearly all six geometric parameters vary to achieve an optimal configuration. Considering that the combined effect of these parameters leads to a significant reduction in drag torque, studying the influence of each individual parameter on drag torque may not be practical. However, some consistent trends appear across all four cases, indicating that certain parameters play a major role in reducing the drag torque. For instance, the distance between the two disks ($h$) increases in all cases compared to the reference configuration. A similar trend is observed for the groove height ($H$) in all cases, with a slight increase in Cases 1 and 2, and a more pronounced increase in Cases 3 and 4. $W$ also increases in Cases 1, 3, and 4, and the same trend is seen for the outer radius (${R}_{2}$). In contrast, the inner radius (${R}_{1}$) and groove angle ($\alpha$) remain relatively unchanged. These consistent trends warrant further discussion to gain deeper physical insight into their effects. This section presents a detailed parametric analysis based on CFD simulations, considering the variation of ${R}_{1},{R}_{2},H,h,W,\alpha$, including physical explanations for their impact on the drag torque and critical speed.

**Fig. 10:**
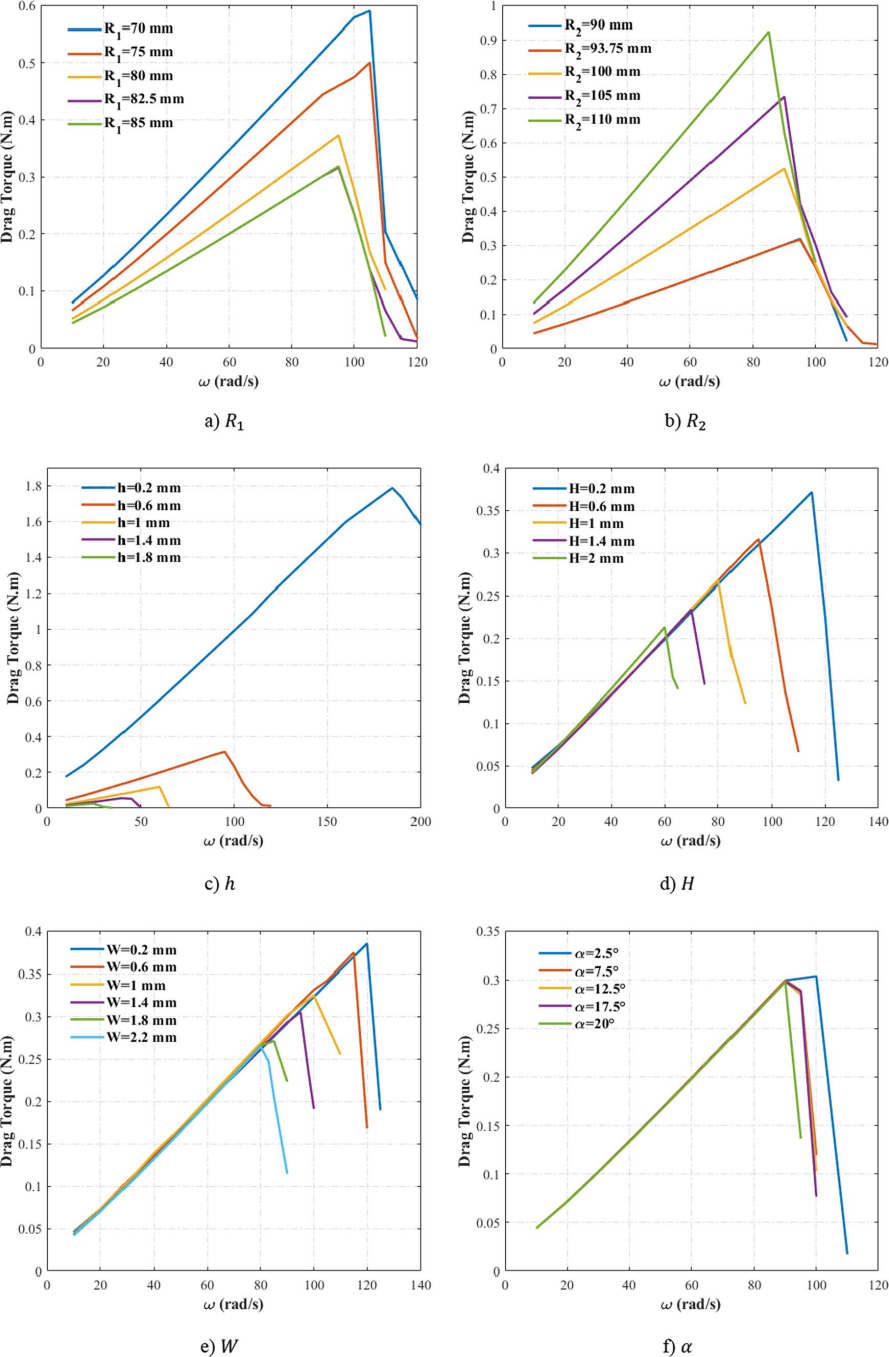
Drag torque—speed curve for cases with various geometric parameters.

Figure 10a illustrates the drag torque variation with rotational speed across different inner radii (70 mm < *R*_1_ < 85 mm), while the other geometric parameters remain constant. Larger ${R}_{1}$ values produce excessively thin clutch designs, while smaller ${R}_{1}$ increases the radial span beyond the practical manufacturing limits. The results demonstrate that increasing ${R}_{1}$ reduces the drag torque and critical speed by approximately 10%. A larger ${R}_{1}$ reduces the clutch disks’ surface area and the difference between the inner and outer radii. This lowers the centrifugal force required for oil passage through the clutch, and causing earlier fluid exit from the flow domain. The resulting empty space becomes filled by entrained air. Furthermore, the reduced contact area between oil and clutch disk directly results in drag torque decrease, as the torque is proportional to the wetted surface area.

**Fig. 11:**
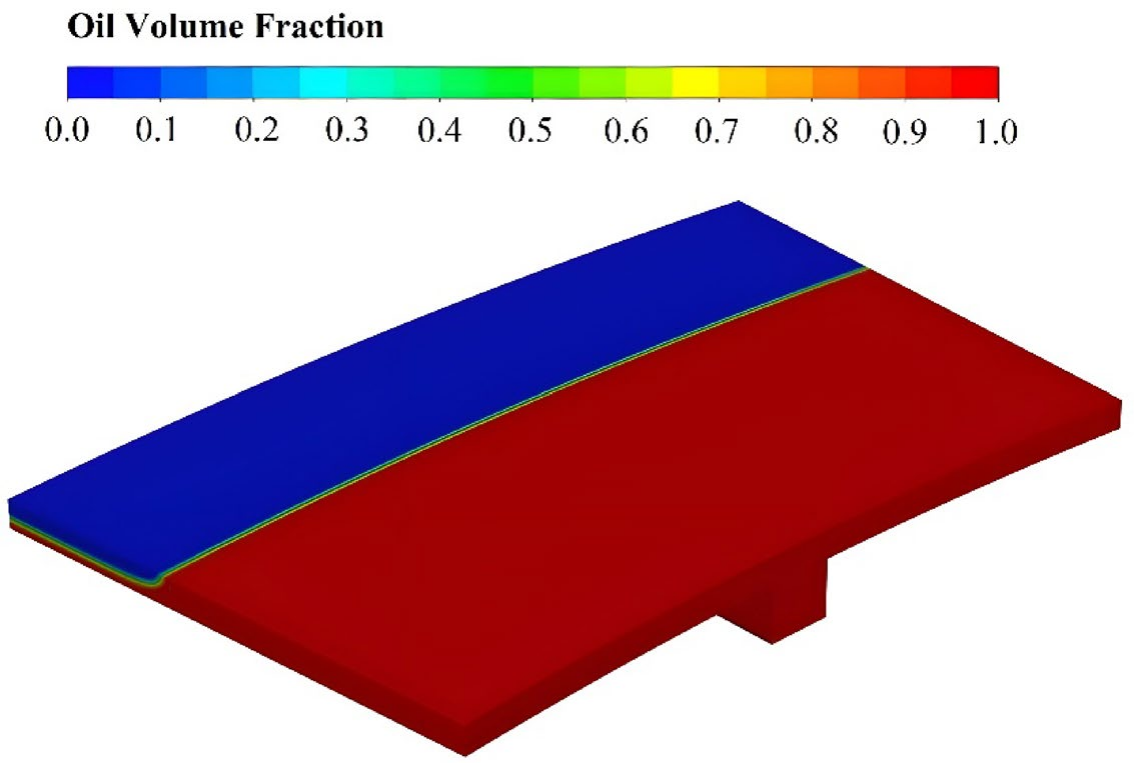
Contour of oil volume fraction through the flow domain for the case with *R*_2_ = 93.75 mm at *ω* = 100 rad/s.

To optimize the geometry of a prefabricated clutch, modifying the outer radius presents a more practical approach than altering the inner radius, since the inner radius is constrained by the fixed dimensions of the gearbox shaft. Figure 10b shows the drag torque-speed curve through different outer radii (90 mm < *R*_2_ < 110 mm). The results show a significant drag torque increase with larger ${R}_{2}$. The maximum drag torque for *R*_2_ = 110 mm is approximately triple the maximum drag torque for *R*_2_ = 90 mm. This aligns with earlier discussions: Greater ${R}_{2}$ expands the oil-disk contact area, elevating drag torque. However, critical speed opposes this behavior unexpectedly and decreases gradually. This behavior likely stems from insufficient oil momentum -with a constant flow rate- at larger radial spans, causing premature aeration near the outer radius due to incomplete oil coverage. Figure 11 confirms this mechanism, illustrating a contour of oil volume fraction for *R*_2_ = 93.75 mm at *ω* = 100 rad/s. It is clear that air filled the domain near outer radius even at low multiphase speeds. Given these two reasons, it can be concluded that variations in the ${R}_{1}$ have a smaller impact on the drag torque compared to changes in the ${R}_{2}$, as confirmed by the optimization results. However, although the optimization results show an opposite trend in the drag torque with respect to ${R}_{2}$ variation, this effect is offset by compensating changes in the other geometric parameters.

Figure 10c illustrates the effect of varying gap between disks ($h$) on drag torque and critical speed while other parameters remain constant. Increasing the gap height ($h$) yields a substantial reduction in both the drag torque and critical speed. For *h* = 0.2 mm, the critical speed reaches 185 rad/s, whereas *h* = 1.8 mm results in ω = 25 rad/s (an 86% decrease). The drag torque follows a similar trend, with a 97% reduction between the mentioned cases. This behavior aligns with the Couette flow dynamics: as established in Ref.^18^. The wet clutch flows are combined from the Couette-type and Poiseuille-type flows, where shear stress—dominantly produced by the Couette-type flow—is inversely proportional to the gap size. This analysis confirms the significant effect of increasing $h$ on the drag torque reduction as evidenced by the optimized configurations.

**Fig. 12:**
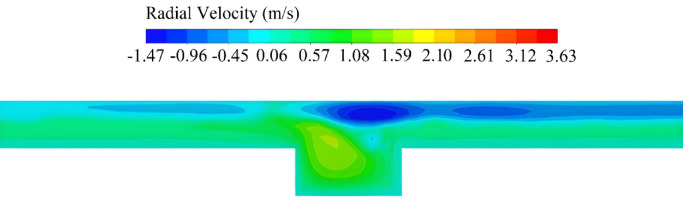
Contour of radial velocity at *R* = *R*_2_ for the case with *H* = 1.2 mm and *ω* = 80 rad/s.

Figure 10d illustrates the drag torque-speed curves for various groove depth ($H$). Increasing $H$ significantly reduces both the drag torque and critical speed. The maximum drag torque at *H* = 2 mm is almost half of that at *H* = 0.2 mm. The critical speed follows a similar trend, where at *H* = 0.2 mm, *ω*_*critical*_ = 115 rad/s, and at *H* = 2 mm, *ω*_*critical*_ decreases by approximately 50% and reaches to 60 rad/s. The radial grooves establish direct flow passages that trap and guide oil outward. This phenomenon accelerates aeration onset and lowers the drag torque because more amount of oil is concentrated into the groove and shear stress over stationary disk (non-grooved disk) will decrease (the drag torque is quantified by integrating the product of radius and shear stress across the stationary disk’s wetted area). This is more visible in Fig. 12, which presents the contour of radial velocity in the outlet boundary of the clutch ($R={R}_{2}$). The velocity contour reveals a positive velocity region in the grooves, demonstrating oil concentration and outward flow toward the clutch exit. Simultaneously, a negative velocity region near the stationary disk confirms air entrainment from the outer radius, where the negative sign indicates inward airflow replacing expelled oil. A similar trend is observed by varying the groove width ($W$) while considering the other variables constant. As shown in Fig. 10e, increasing $W$ reduces both the drag torque and critical speed, though its influence is somewhat weaker than variations in $H$. For instance, increasing $W$ from 0.2 to 2.2 mm reduces the maximum drag torque by 31%. These results demonstrate that larger groove dimensions consistently improve drag loss performance. This is fully consistent with the observations from the optimized cases. However, practical constraints prevent excessive groove geometry enlargement. Key limitations include minimum clearance requirements between the clutch pairs, increased weight and volume of grooved disks, and manufacturing challenges at extreme dimensions.

Figure 10f demonstrates the influence of groove angle on the drag torque-speed characteristics, indicating that critical speed decreases with increasing angle. However, the drag torque shows minimal variation across angles, with only a 2% reduction between $\alpha =0^\circ$ and $\alpha =20^\circ$. As observed in the optimized configurations, variations in the groove angle have little impact on the drag torque. These results indicate that groove cross-section geometry exert a greater influence on drag torque reduction than groove angle.

It should be noted that the trends observed in the parametric study represent the isolated influence of each parameter, whereas the optimization accounts for the coupled variation of all geometric features. For example, the parametric results show that decreasing the outer radius ${R}_{2}$ tends to reduce drag torque. However, in three out of four optimized cases ${R}_{2}$ increased relative to the reference design (Table 5). This apparent discrepancy arises from compensating effects: the increase in ${R}_{2}$ was counterbalanced by simultaneous modifications in other parameters such as gap clearance $h$ and groove depth $H$, which have a more dominant role in reducing drag torque. This finding underscores a critical point: while the parametric study provides essential physical insight into the role of individual parameters, the optimization highlights that the best-performing designs emerge from the interplay between parameters rather than from changes in any single parameter alone.

## Conclusion

This study aims to conduct a geometric optimization of a wet clutch with radial grooves. To this end, the predictive capability of the recently developed analytical model by Sadafi et al.^18^ was first evaluated through comparison with pre-validated numerical simulations. The comparative analysis showed that the analytical model reliably captures the drag torque trends across variations in geometric parameters. The comparisons demonstrate reasonable accuracy, with a maximum deviation of 8% from the numerical results. Considering the high computational cost of CFD simulations, the analytical model was subsequently used to generate a database for training data-driven models and conducting optimization studies.

Two databases, each comprising 600 samples, were generated using the Sobol sampling method. The second database incorporated dynamic viscosity as an additional input parameter alongside geometric variables to enhance model accuracy. Artificial neural network models were developed using MATLAB software and trained on both datasets. The trained models exhibited excellent performance, with coefficient R^2^ values approaching unity in both cases and MSE values on the order of 10^−4^.

Subsequently, the optimization study was conducted using the trained ANNs, employing a Genetic Algorithm implemented in MATLAB. Four distinct cases were examined, differentiated by their objective functions (with or without $\mu$ as an input) and constraints on the inner radius (${R}_{1}$) imposed by practical design considerations. To validate the optimization results, fluid flow simulations were performed for all optimized configurations, with the outcomes compared against reference case drag torque. All four cases demonstrated substantial improvements, achieving at least a 70% reduction in both drag torque and critical speed relative to the reference configuration. These results confirm the effectiveness of the optimization approach in significantly decreasing the clutch’s drag loss characteristics.

Finally, to interpret the optimization results and develop a comprehensive understanding of the geometric effects, a parametric study was conducted using CFD. The simulations examined the effect of six geometric parameters variations on the drag torque. The numerical results indicate that drag torque reduction and earlier aeration onset are achieved through the following geometric modifications: (1) increased groove depth, (2) increased groove width, (3) increased clearance between disks, (4) increased inner radius, (5) increased groove angle, and (6) decreased outer radius. Parametric analysis reveals that the clearance between disks has the most pronounced effect on the drag torque reduction, while the groove angle demonstrates the least influence. The obtained results were in close agreement with the optimization findings and provided justification for the corresponding geometric variations.

Finally, it is important to emphasize that the optimized geometries obtained in this study represent theoretical optima that may face limitations in practical implementation. For example, the inter-disk clearance is constrained in real clutch designs: excessively large gaps increase the axial size and weight of the clutch assembly, introduce the risk of interference with adjacent plates in multi-disk packs, and reduce the torque transmission capacity during engagement. Similarly, extremely small groove angles may be challenging to machine with accuracy in industrial practice. These considerations highlight that while the optimization framework identifies valuable design trends, the translation to manufacturable designs requires balancing drag-torque reduction with structural integrity, torque capacity, and production feasibility.

Building on this work, several potential topics are suggested. The optimization framework could be reapplied to experimental test data to validate its robustness under real-world conditions. Alternative groove geometries—such as waffle-shaped, sun-burst, or conical patterns—can be examined through combined numerical and experimental studies. The fluid temperature variation effects on the drag loss can be incorporated into both the data-driven modeling and optimization processes. Recent hydrodynamic studies suggest that curved groove patterns aligned with flow streamlines may enhance performance; this hypothesis merits dedicated numerical and experimental investigation. Additionally, the analytical model proposed by Sadafi et al.^18^ could be refined by calibrating its parameters against high-accuracy test data and machine learning outputs, potentially improving its predictive accuracy for industrial applications.

## Data Availability

The datasets and codes analyzed or used during the current study are available from the corresponding author upon reasonable request.
